# Postural control impairments following neurotoxic chemotherapy in women with cancer: a prospective observational study

**DOI:** 10.1186/s12905-025-03790-4

**Published:** 2025-05-30

**Authors:** Aline Reinmann, Alexandre Bodmer, Thibaud Koessler, Joseph Gligorov, Anne-Violette Bruyneel

**Affiliations:** 1 Geneva School of Health Sciences, Physiotherapy Department, HES-SO University of Applied Sciences and Arts Western Switzerland, Rue des Caroubiers 25, Carouge, Geneva, CH-1227 Switzerland; 2 Sorbonne University, INSERM, Centre de Recherche Saint Antoine, CRSA, Paris, F-75012 France; 3 Service of Oncology, Geneva University Hospitals, Geneva, Switzerland; 4 University of Geneva, Geneva, Switzerland; 5 Department of Oncology, AP-HP, Sorbonne University, Tenon Hospital, Paris, France

**Keywords:** Breast cancer, Chemotherapy, Postural control, Peripheral neuropathy

## Abstract

**Purpose:**

This study aimed to compare changes in postural control in different testing conditions involving sensory disturbances, quality of life and neurotoxicity concerns in women with cancer before and after chemotherapy with taxanes. The second aim was to compare postural control between chemotherapy-induced peripheral neuropathy (CIPN) severity subgroups.

**Method:**

The 33 participants with breast cancer (age 48.15 ± 9.88 years) were tested during the month preceding (baseline) and the month after the end of chemotherapy. Postural control was assessed on a force platform (100 Hz) in different conditions: eyes open/closed, rigid/foam surface, with/without tendon vibration, dual task, and limits of stability. CIPN severity and neurotoxicity concerns were also evaluated. Paired t-tests or Wilcoxon sign rank tests were used, and a Benjamini-Hochberg correction was applied.

**Results:**

After chemotherapy, greater postural adjustments were required to maintain balance in the reference condition (*p* ≤ 0.02), and in conditions with visual (*p* ≤ 0.02), foam (*p* ≤ 0.04), and dual task disturbances (*p* ≤ 0.01), but not in the vibration and limits of stability conditions. No difference was found between the CIPN subgroups. The neurotoxicity score worsened after chemotherapy (-8.61 ± 7.53, *p* ≤ 0.01).

**Conclusions:**

Postural control was impaired after chemotherapy, particularly in conditions with visual disturbances and dual task. Anteroposterior center of pressure displacements with mediolateral ground reaction forces were particularly increased.

**Clinical implications:**

Systematic assessment of postural control with eyes closed in women with breast cancer would help to identify chemotherapy-induced postural control disorders and determine treatment needs.

**Trial registration:**

Clinical Trial Registration number NCT04692168. Registration date: 28.12.2020

**Supplementary Information:**

The online version contains supplementary material available at 10.1186/s12905-025-03790-4.

## Introduction

Postural control [1], defined as the ‘act of maintaining, achieving or restoring a state of balance during any posture or activity’ [2], can be altered by chemotherapy. The alterations in postural control could be related to chemotherapy-induced peripheral neuropathy (CIPN) found in over 60% of patients treated with taxanes [3]. CIPN causes pain, paresthesia, dysesthesia, impaired sensation, increased vibratory and reflex sensitivity, and loss of nerve conduction in the lower limbs, which could prevent adequate postural adjustment [4].

Several studies previously found impairment of quasi-static balance in people treated with chemotherapy compared to matched healthy individuals [5–10]. The increase in speed, length, root mean square (RMS), and area of center of pressure (CP) parameters demonstrated an increase in energy expenditure and overcorrection of the CP to maintain balance [11, 12] as well as a move closer to the limits of stability [12]. Three longitudinal studies [12–14] that took baseline postural parameters into account showed a decrease in postural control from the first cycle of taxane chemotherapy, which persisted beyond the end of the treatment [12, 13].

Postural control disorders induced by chemotherapy lead to the development of coping strategies. Previous studies found that visual perturbations accentuated postural control impairment post chemotherapy, suggesting an upweighting of visual information because of a deficient somatosensory system [1, 4, 15]. However, the small number of prospective studies with different sensory test conditions limits the understanding of the evolution of postural control and coping strategies after chemotherapy. Studies that are specific to one cancer type and treatment administered [1], and that integrate different testing conditions, with perturbations of different sensory inputs through various means [4] are required. Adaptation strategies implemented by individual can be assessed with eyes shut to test the influence of the visual system and standing on foam or using tendon vibration to test the influence of the somatosensory system [16, 17]. Dual-task and dynamic stability assessments can complement the evaluation by resembling daily life situations [18, 19].

Measurement of CP and ground reaction force (GRF) parameters using a force platform would allow changes in postural adjustments and postural muscle actions to be precisely determined and would provide further insights into compensatory strategies used to maintain balance. The CP corresponds to the point of application of the mean reaction force and provides an indication of postural adjustments in the horizontal plane [11], whereas the ground reaction force indicates the resulting dynamics of postural muscle activation in the three planes [20]. These parameters are thus complementary and CP and GRF data would help to determine appropriate evaluations and the need for a systematic assessment of postural control after chemotherapy. Postural control deficits increase the fear of falling [21] that may lead to deconditioning and difficulties in daily living activities impacting the person’s autonomy and quality of life [22].

This study had three aims. (1) To compare the quasi-static and dynamic postural control ability of women with cancer before and after chemotherapy in different conditions of postural perturbation to identify underlying sensorimotor dysfunctions and coping strategies. We hypothesized that postural control would be reduced post chemotherapy, particularly in visual disturbance conditions, because of the decrease in sensory feedback caused by somatosensory deficits. (2) To investigate quality of life, specifically neurotoxicity concerns, after chemotherapy. We hypothesized that neurotoxicity score would be increased post chemotherapy because of CIPN. (3) To determine the association between CIPN severity (mild, moderate, or severe) and the severity of impairment of postural control, dynamic balance and neurotoxicity concerns. We hypothesized that postural control, dynamic balance and neurotoxicity score would be negatively impacted by the CIPN severity.

## Methods

### Study design

This observational prospective study took place in the Geneva University Hospitals from October 2021 to December 2022. The study reporting followed the STROBE guidelines.

### Participants

We included women aged 18 to 65 years with gynecological or breast cancer who were about to start neurotoxic chemotherapy (paclitaxel, docetaxel, nab-paclitaxel or paclitaxel-carboplatin) and who could stand upright for 30s without assistance. Women with pre-existing pathologies altering postural control like diabetes, previous exposure to known neurotoxic chemotherapy, or an inability to perform the tests because of difficulties in comprehension or psychological problems were excluded.

The participants were consecutively recruited during a medical appointment. After consenting to be contacted, a research assistant not involved in their routine care reached the participant and provided written and oral information about the study (nature, purpose, procedure, risks, and benefits). The participant had at least 24-hours reflection time before giving written consent and starting the study procedure. The study was approved by the Geneva ethics committee (2020 − 01639).

### Study procedure

Participants completed two assessments: one within one month before the start and one within one month after the end of chemotherapy. The chemotherapy lasted about 12 weeks. After collecting demographic and lifestyle data, postural control was assessed using a force platform, CIPN using the modified Total Neuropathy Score (mTNS), quality of life and neurotoxicity using the Functional Assessment of Cancer Therapy/Gynaecologic Oncology Group Neurotoxicity (FACT/GOG-NTX). The tests were conducted by a specifically trained physiotherapist in a quiet room.

### Test descriptions

*Quasi-static postural control* was measured using a 600 × 500 mm force platform at 100 Hz (Biomechanical Force Plate Systems type 9260AA6, Kistler Instrument AG, Winterthur, Switzerland), a valid [23] and reliable tool [24]. The participants were instructed to stand as still as possible for 30s [24], arms at their side, heels 10 cm apart and halluces 15 cm apart [5], looking at a fixed point on a wall at eye level at a distance of 0.9 m [25]. The procedure was repeated three times per condition with 10s rest between the trials [24]. The test was conducted under different conditions performed randomly: on rigid surface with eyes open (EO) and closed (EC), on foam surface with eyes open (EOF) and closed (ECF), with vibration eyes open (EOV) and closed (ECV), with dual task (DT). For the foam conditions, a 48 × 40 × 6 cm piece of foam was used to modify proprioceptive feedback (Airex, Airex AG, Sins, Switzerland) [16]. For the vibration conditions, vibrators (Vibrasens, Technoconcept, Manosque, France) with a 80 Hz frequency and 0.5 mm amplitude [26] were positioned on the Achilles tendon to disturb somatoproprioceptive feedback [27]. The cognitive task involved participants writing an unusual text message on a smartphone while maintaining balance. Since postural control is a constant search for equilibrium and reflects a continuous process [28], the term ‘quasi-static’ has been preferred to the term ‘static’ postural control.

*Dynamic balance* was tested with the limits of stability by asking participants to lean as far as possible in four directions (anterior, posterior, right and left) three times without bending their hips or lifting either foot off the platform [29]. Two learning trials were conducted prior to the test to avoid learning bias [30].

The changes in center of pressure (CP) and ground reaction forces (GRF) were extracted for all conditions. We calculated the mediolateral (ML) root mean square (RMS), the total RMS, the anteroposterior (AP) RMS, the CP area, the total length, the AP and ML lengths, and the maximal velocities [31]. The selected GRF parameters were AP, ML, and vertical (V) maximum peak values, and force variability.

*CIPN* were assessed using the mTNS (range 0–24). This included person-reported sensory and motor symptoms, sensation, vibratory sensitivity, strength and tendon reflexes [32]. Each component is individually scored between 0 and 4 and the sum of the components gives the final score. A score below 9 indicates mild CIPN, between 9 and 16 moderate CIPN, and above 16 severe CIPN [33]. More information is provided in the previously published protocol [34]. This tool has excellent validity (*r* = 0.99 compared to TNS) [32] and intra-rater reliability (ICC = 0.99) [35].

*Quality of life and neurotoxicity* were assessed using the FACT/GOG-NTX questionnaire which measures physical, social/family, emotional and functional well-being, and neurotoxicity concerns on a 4-point Likert Scale (range 0-152). Sub-scores and total score were calculated, with higher scores indicating better quality of life and lesser neurotoxicity concerns [36]. A good internal consistency (Cronbach’s alpha > 0.8) [37] and test-retest reliability (ICC = 0.84) [38] as well as the validation in French [39] support its use in practice.

### Statistical analysis

*Sample size calculation* was performed using the G*Power V.3.1 software package and a two-sided *t*-test for paired data (HHU Düsseldorf, Germany, 2017) [40]. With the significance level set at 0.05, a power of 90%, and an effect size of 0.61 [12], 31 participants were required for this study. A 16% drop-out was anticipated [12], which required 37 participants to be included.

*Data processing* of all CP data from the postural control tests was performed using a fifth-order low-pass Butterworth filter at 30 Hz. Then, the mean of the three trials was calculated for all postural control tests. For the limits of stability, the AP and ML, CP length deltas were normalized using the formula: CP parameter (mm) / mean size of right and left feet (mm). Romberg (eyes closed / open) and proprioceptive (foam / rigid surface) ratios were calculated for CP total length [41]. Vibration ratios (vibration / non vibration) were also calculated. The V GRF was normalized to the participant’s bodyweight (V GRF (kg) / body weight (kg)).

Categorical variables are expressed as frequencies and continuous variables are expressed as means and standard deviations.

A modification of the initial statistical analysis plan was necessary because the initially planned linear mixed models [34] did not fulfil application conditions (residues’ homoscedasticity could not be confirmed). After verifying the distribution of the CP and GRF deltas, paired *t*-tests were used to assess the change in the CP and GRF parameters pre- and post-chemotherapy in each condition (EO, EC, EOF, ECF, EOV, ECV, and DT). In the case of non-normal distribution, a Wilcoxon sign rank test was used. To account for the number of *t*-tests, a multiplicity correction was applied. This correction was based on Benjamini Hochberg’s formula using an online calculator (https://www.sdmproject.com/utilities/?show=FDR). The same procedure was used for dynamic standing balance. Paired *t*-tests were used to assess changes in FACT/GOG-Ntx total and sub-scores. The association between CIPN severity and the pre-post change in CP parameters in EC and ECF conditions was tested using a Mann Whitney test for each severity subgroup (mild vs. moderate, no participant had severe CIPN). For all analyses, a two-tailed *p*-value < 0.05 was considered significant. The analyses were conducted using Stata software (v.15, Stata Corporation, USA).

## Results

### Study completion and socio-demographic characteristics

Participants were recruited from October 2020 to July 2022. Mean inclusion rate per week was 6%. Most reasons for refusal were side-effects of cancer and treatments (i.e. fatigue), already having too many medical appointments, and not wishing to participate. In total, 35 participants were included (Table 1). They all gave informed consent to participate in the study. The high proportion of breast cancer was due to the greater representation of patient with breast cancer in the study unit and the treatment protocol that facilitated their inclusion (longer delay between the first appointment and the start of neurotoxic chemotherapy compared with gynecological cancers). Neurological comorbidities concerned distal upper extremity problems.

**Table 1:** Demographic characteristics. Data are mean ± sd (Min-Max) or N (%). BMI = body mass index, ec = epirubicin and cyclophosphamide, tnm = tumor, lymph nodes, metastasis

Demographic characteristics (*N* = 35)		
Characteristics	Values	
**Age (years)**	48.71 ± 9.86 (28–65)	
**Height (cm)**	163.44 ± 5.61 (153–175)	
**Weight (kg)**	68.01 ± 13.17 (42–90)	
**BMI (kg/m** ^**2**^ **)**	25.49 ± 5.03 (16.82–35.25)	
**Comorbidities, n (%)**		
None	8 (25)	
Respiratory	4 (11)	
Cardiovascular	3 (9)	
Musculoskeletal	11 (31)	
Dermatology	3 (9)	
Digestive	9 (26)	
Neurological	3 (9)	
Endocrine/metabolic	6 (17)	
Psychiatric	7 (20)	
**Cancer type, n (%)**		
Breast	34 (97)	
Uterus	1 (3)	
**TNM classification (** ***n*** ** = 34)**		
T0	4	
T1	11	
T2	14	
T3	4	
T4	1	
N0	18	
N1	10	
N2	3	
N3	3	
M0	31	
M1	3	
**Treatment**		
EC + Paclitaxel	21	
Paclitaxel	2	
EC + Paclitaxel changed for Docetaxel	3	
Paclitaxel changed for Nab-Paclitaxel	2	
EC + Paclitaxel + Carboplatin	4	
Cyclophosphamide + Docetaxel	2	
Paclitaxel + Carboplatin	1	
**Treatment change, n (%)**	5 (14)	
**Smoking**		
Smoked in the past, n (%)	19 (54)	
Current smoker, n (%)	11 (31)	
Pack-year (*n* = 19)	16.93 ± 11.17 (3–40)	
**Alcohol**		
Consumption (mean glasses of alcohol / week)		1.62 ± 4.87 (0–28)
**Physical activity**		
From 3 to 6 MET (hour / week)	10.04 ± 5.59 (1–28)	
Above 6 MET (hour / week)	0 ± 0 (0–0)	

Two participants did not complete the second assessment for logistical reasons or because they had too many medical appointments so 33 completed the whole procedure (age 48.15 ± 9.88 years, height 163.89 ± 5.40 cm, weight 68.26 ± 13.14 kg, 100% breast cancer). Assessments took place 14 ± 19 days before and 15 ± 11 days after the neurotoxic chemotherapy. Ten (30%) participants had a treatment change (early discontinuation or change of treatment) because of CIPN. Thirteen participants (39%) progressed from CIPN grade 1 to 2 after chemotherapy (*supplementary material 1*). Twenty-five participants (76%) had a difference in NTx score greater than three points, considered to be the minimal clinical important change [42]. A total of twenty-seven participants (82%) had evidence of CIPN (either treatment change, grade 2 mTNS, or NTx score change ≥ 3). Fatigue score post assessment was higher by 16.06 ± 29.68 units on a VAS (/100), *p* = 0.019 at the second assessment.

Errors in the handling of the platform lead to six missing values for ML and AP GRF. One participant was excluded from the ML and AP GRF analyses and two from the V GRF analyses due to outliers that did not correspond to the actual CP values for the conditions performed.

### Comparison between baseline and post-treatment quasi-static balance in each condition

**Graph 1:**
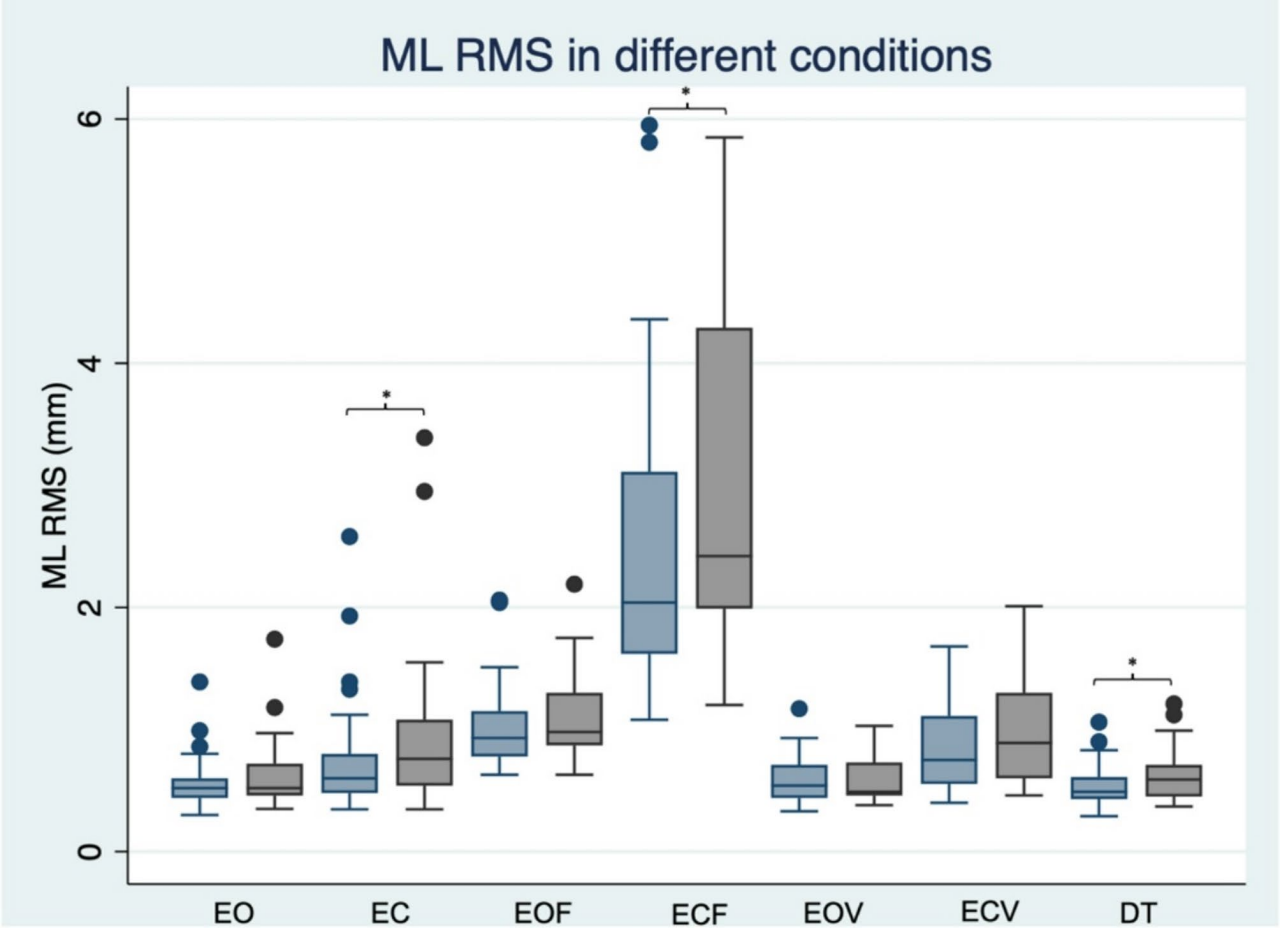
Box plot of ML RMS in different conditions. In blue, results pre chemotherapy, in grey, results post chemotherapy. E = eyes, C = closed, DT = dual task, F = foam, ML = mediolateral, RMS = root mean square, V = vibration. *n* = 33

*In EO condition*, total and AP CP lengths and RMS were increased after chemotherapy (*p* < 0.02) whereas the ML length and RMS, the area, and the maximum speed were not significantly different (Table 2; Graph 1). For GRF parameters, ML maximum peak values and variability, AP variability, and V maximum peak values were increased (*p* < 0.02) whereas the AP maximum peak values, and the V variability were not significantly different (*p* > 0.05).

**Table 2:** CP and GRF parameters. Data are mean ± sd (Min-Max). *n* = 33 for CP, *n* = 24 for ML and AP GRF, *n* = 31 for V GRF. AP = anteroposterior, cp = center of pressure, grf = ground reaction force, ML = medio-lateral, v = vertical. Corrected p-values

Eyes open condition (EO)					Eyes closed condition (EC)				Eyes closed on foam condition (ECF)			
	**Pre**	**Post**	**Difference**	***p-value***	**Pre**	**Post**	**Difference**	***p-value***	**Pre**	**Post**	**Difference**	***p-value***
**CP**												
Planar												
Length (mm)	380.73 ± 124.63 (192.01–834.58)	420.81 ± 132.24 (268.39–923.50)	40.08 ± 79.59 (-157.91–224.86)	**0.022**	492.35 ± 223.10 (298.14–1340.21)	608.95 ± 259.14 (308.80–1548.88)	116.60 ± 123.73 (-246.21–407.19)	**<0.001**	1472.92 ± 674.08 (736.36–3154.51)	1877.99 ± 830.16 (801.16–3893.56)	405.08 ± 420.40 (-386.39–1357.69)	**<0.001**
Area (mm^2^)	237.67 ± 236.27 (53.26–1023.84)	258.74 ± 220.88 (52.52–993.71)	21.06 ± 134.11 (-389.05–420.22)	0.460	343.36 ± 421.84 (59.92–2292.99)	496.68 ± 371.98 (59.92–1330.67)	153.32 ± 330.80 (-1243.11–1015.41)	**0.013**	1991.21 ± 1311.29 (670.11–6209.70)	2624.86 ± 1498.59 (945.13–7032.63)	633.66 ± 857.80 (-1091.41–3056.10)	**<0.001**
Maximal speed (mm/s)	67.96 ± 27.89 (31.96–196.44)	72.92 ± 28.66 (39.14–177.75)	4.96 ± 20.02 (-23.41–71.33)	0.292	88.25 ± 39.63 (39.50–6.48)	108.29 ± 50.23 (42.99–260.66)	20.03 ± 27.94 (-25.70–89.09)	**0.001**	254.61 ± 141.70 (107.58–701.67)	324.29 ± 154.46 (142.76–653.46)	69.68 ± 95.35 (-159.81–308.99)	**<0.001**
RMS (mm)	0.78 ± 0.32 (0.44–2.18)	0.89 ± 0.36 (0.51–2.38)	0.10 ± 0.17 (0.23–0.57)	**0.011**	1.08 ± 0.53 (0.56–2.95)	1.41 ± 0.73 (0.58–3.80)	0.32 ± 0.35 (-0.12–1.63)	**<0.001**	3.38 ± 1.71 (1.48–7.73)	4.33 ± 2.05 (1.81–9.31)	0.95 ± 1.15 (-1.62–3.89)	**<0.001**
ML												
Length (mm)	203.67 ± 73.06 (97.87–485.12)	221.02 ± 83.64 (193.51–632.23)	17.35 ± 47.86 (-106.25–128.35)	0.104	265.54 ± 142.45 (133.51–839.95)	313.11 ± 184.00 (133.51–1046.55)	47.56 ± 96.93 (-219.37–386.10)	**0.009**	831.32 ± 368.26 (405.23–1825.44)	1019.27 ± 422.12 (430.06–1944.89)	187.95 ± 202.30 (-281.76–580.15)	**<0.001**
RMS (mm)	0.56 ± 0.21 (0.3–1.39)	0.61 ± 0.27 (0.35–1.74)	0.06 ± 0.15 (0.25–0.38)	0.102	0.77 ± 0.46 (0.35–2.58)	0.94 ± 0.66 (0.35–3.39)	0.17 ± 0.39 (-0.63–1.83)	**0.019**	2.48 ± 1.23 (1.08–5.95)	3.02 ± 1.33 (1.2–5.85)	0.53 ± 0.75 (-1.59–1.96)	**<0.001**
AP												
Length (mm)	277.52 ± 89.08 (145.64–577.38)	310.45 ± 91.07 (193.51–632.23)	32.93 ± 55.88 (-98.12–156.44)	**0.011**	356.57 ± 144.84 (222.08–867.33)	452.46 ± 164.67 (235.68–926.06)	95.89 ± 84.00 (-73.99–261.58)	**<0.001**	1035.02 ± 493.26 (525.10–2304.64)	1362.84 ± 638.88 (590.49–3122.63)	327.82 ± 336.86 (-383.53–1146.73)	**<0.001**
RMS (mm)	0.77 ± 0.30 (0.41–2.05)	0.86 ± 0.31 (0.49–2.06)	0.10 ± 0.17 (0.22–0.52)	**0.011**	1.02 ± 0.43 (0.57–2.42)	1.31 ± 0.54 (0.60–2.77)	0.30 ± 0.26 (-0.04–1.14)	**<0.001**	3.08 ± 1.55 (1.47–6.79)	4.04 ± 1.93 (1.75–9.26)	0.97 ± 1.09 (-1.24–3.84)	**<0.001**
**GRF**												
V												
Max values (kg)	9.75 ± 0.25 (9.14–10.34)	10.08 ± 0.59 (8.77–11.29)	0.33 ± 0.53 (-0.95–1.49)	**0.007**	9.76 ± 0.26 (9.16–10.36)	10.08 ± 0.59 (8.77–11.27)	0.34 ± 0.51 (-0.69–1.59)	**0.009**	10.35 ± 0.44 (9.52–11.50)	10.91 ± 0.80 (9.37–13.39)	0.56 ± 0.65 (-1.21–1.88)	**0.004**
Variability (kg)	0.01 ± 0.00 (0.01–0.02)	0.01 ± 0.01 (0.01–0.04)	0.00 ± 0.00 (-0.01–0.01)	0.746	0.01 ± 0.01 (0.01–0.08)	0.01 ± 0.00 (0.01–0.03)	0.00 ± 0.01 (-0.02–0.01)	0.122	0.11 ± 0.08 (0.03–0.32)	0.15 ± 0.11 (0.04–0.40)	0.04 ± 0.05 (-0.05–0.16)	**0.004**
ML												
Max values (kg)	0.30 ± 1.21 (-0.93–5.43)	0.81 ± 1.80 (-0.45–8.01)	0.50 ± 0.91 (-0.82–2.90)	**0.023**	1.18 ± 1.93 (-1.02–6.25)	2.32 ± 3.21 (-0.49–12.08)	1.14 ± 2.09 (-1.24–9.13)	**0.016**	8.36 ± 4.32 (2–15.13)	11.38 ± 6.14 (3.09–26.13)	3.02 ± 3.59 (-2.05–11.21)	**0.006**
Variability (kg)	0.53 ± 0.41 (0.22–2.32)	0.67 ± 0.54 (0.24–2.86)	0.14 ± 0.21 (-0.08–0.79)	**0.007**	0.77 ± 0.52 (0.27–2.21)	1.05 ± 0.77 (0.24–3.16)	0.28 ± 0.43 (-0.09 − 1.91)	**0.006**	2.91 ± 1.26 (1.06–5.11)	3.59 ± 1.57 (1.36–7.55)	0.68 ± 0.75 (-0.81–2.44)	**0.004**
AP												
Max values (kg)	4.61 ± 2.61 (1.88–15.39)	5.57 ± 3.81 (2.25–20.06)	0.96 ± 3.83 (-4.30–17.42)	0.164	5.94 ± 3.19 (2.63–14.93)	6.88 ± 3.40 (2.79–18.32)	0.95 ± 2.77 (-5.06–10.20)	0.100	15.46 ± 7.40 (5.37–37.9)	18.45 ± 8.60 (6.77–42.47)	2.99 ± 6.88 (-13.41–24.14)	0.066
Variability (kg)	0.75 ± 0.59 (0.30–3.26)	0.92 ± 0.61 (0.36–3.38)	0.17 ± 0.16 (-0.04–0.67)	**<0.001**	1.06 ± 0.58 (0.38–2.79)	1.50 ± 0.82 (0.55–3.68)	0.44 ± 0.39 (-0.03–1.80)	**<0.001**	3.68 ± 1.65 (1.36–8.02)	4.82 ± 2.07 (1.93–9.08)	1.15 ± 1.32 (-1.77–4.51)	**0.004**

*In EC condition*, all CP and GRF parameters were increased after chemotherapy (*p* < 0.02) except for the GRF AP maximum peak values, and V variability (Table 2).

*In EOF condition*, the total and AP CP lengths, the maximal CP speed, and the total and AP CP RMS were increased. Except for the AP maximum peak GRF, all the GRF parameters were increased (*p* < 0.04, *supplementary material 1*).

*In ECF condition*, all CP and GRF parameters were increased after chemotherapy (*p* < 0.01, Table 2).

*In EOV and ECV conditions*, no differences in CP and GRF parameters were found. Only the V maximum peak GRF was significantly increased (*p* = 0.02 for EOV and 0.04 for ECV).

*In DT condition*, all CP and GRF parameters were increased except the maximal speed, the AP maximum peak GRF, and the V variability (*p* > 0.12, *supplementary material 1*).

The Romberg and the Romberg on foam increased from 1.29 to 1.45 and 2.46 to 2.79 (*p* < 0.01), respectively. The proprioceptive ratios did not change (1.60 to 1.62, *p* = 0.62 with eyes open and 3.06 to 3.15 with eyes closed, *p* = 0.50). The vibration ratios changed from 1.09 to 1.02 with eyes open and 1.31 to 1.14 with eyes closed (*p* < 0.01).

#### Comparison between baseline and post-treatment CP and GRF parameters in the dynamic standing balance condition

For the limits of stability, CP and GRF parameters did not change post chemotherapy, except for the V maximum GRF, which increased (*p* < 0.01).

#### Comparison between baseline and post-treatment quality of life and neurotoxicity with the FACT/GOG-Ntx

Only the neurotoxicity subscale was impaired after the chemotherapy (-8.61 ± 7.53, -23%, *p* < 0.01, Table 3).

**Table 3:** FACT/GOG-Ntx results. Data are mean ± sd (Min-Max). Corrected p-values

FACT/GOG-Ntx questionnaire (*n* = 33)				
	**Pre**	**Post**	**Difference**	***p-value***
Physical well-being (/28)	19.27 ± 5.90 (7–28)	17.21 ± 5.52 (9–28)	-2.06 ± 6.29 (-15–10)	0.585
Social / familial well-being (/28)	22.02 ± 4.24 (10–28)	21.65 ± 4.47 (6–26.83)	-0.36 ± 4.10 (-9–9)	0.201
Emotional well-being (/24)	18.36 ± 3.74 (8–24)	18 ± 3.86 (9–24)	-0.36 ± 3.72 (-9–7)	1.000
Functional well-being (/28)	16.46 ± 5.34 (7–25)	15.52 ± 4.96 (5–26)	-0.94 ± 4.96 (-10–10)	1.000
Neurotoxicity subscale (/44)	38.18 ± 6.60 (16–44)	29.58 ± 9.66 (9–44)	-8.61 ± 7.53 (-25–3)	**≤0.001**
Total	114.29 ± 19.10 (68–141)	101.96 ± 20.18 (75–141)	-12.33 ± 18.34 (-48–15.83)	**0.035**

#### Comparison between subgroups of CIPN severity (mTNS)

No differences between the pre-post change in postural control of the mild and moderate severity subgroups were found in the EC, ECF, and limits of stability conditions for any parameters except the GRF AP variability in the EC condition for which the value of the moderate severity subgroup was higher than the one of the mild severity subgroup (+ 0.38 kg, *p* = 0.04, *supplementary material 1*). Only changes in the neurotoxicity subscale score were accentuated in the moderate vs. mild CIPN subgroup (+ 5.73, *p* = 0.04).

## Discussion

This study found a reduction in postural control after chemotherapy in women with breast cancer, shown by an increase in CP and GRF parameter values. The difference was greatest in the conditions with visual or dual task perturbation, confirming our hypothesis. Quasi-static balance in the vibration condition, and dynamic standing balance were unchanged after chemotherapy. The neurotoxicity subscale score was poorer after chemotherapy. Contrary to our hypothesis, no difference in postural control parameters was found between the two subgroups of CIPN severity.

The reduction in postural control post chemotherapy in the reference condition (EO) as well as in the conditions with sensory disturbances (EC, EOF, ECF, DT) is consistent with other studies [12–14]. As in previous work [12, 14], multidirectional instabilities were observed with CP and GRF parameters providing complementary information on changes in postural adjustments and efforts made to maintain balance. While CP parameters changed mainly in the total and AP directions, GRF max changed mainly in the ML plan. This may indicate an increased AP correction of the CP to keep the center of mass projection as close as possible to the base of support [43], with a greater ML hip muscle activity to maintain balance [44] after chemotherapy.

The different testing conditions revealed more postural control impairments after chemotherapy in conditions with visual perturbances (EC, ECF). In these conditions, almost all parameters deteriorated, in line with previous work [12, 14]. The greater instabilities when visual input was disturbed may be explained by a reweighting of sensory information with increased use of visual and vestibular inputs following chemotherapy [1]. This coping strategy hypothesis is supported by the increase in the Romberg ratios on rigid and foam surfaces as well as the increased postural instabilities in DT condition after chemotherapy, where visual inputs could not be used for stabilization.

Foam conditions (EOF, ECF) demonstrated strong muscle activity to maintain balance both pre and post chemotherapy with high ML and V GRF max and variability. Foam seemed to act as a mechanical perturbator of the somatosensory system that enhanced the effort of standing and required more active neural control [20, 45, 46]. After chemotherapy, postural instabilities were found on foam with eyes open and closed.

In contrast, in vibration conditions (EOV, ECV) no post-chemotherapy loss of postural control was found. Disruption of Ia fibers induced by molecular and cellular dysfunctions after chemotherapy [47] could have limited the impact of the vibration. Taxanes slow the feedback transmitted by Ia fibers and reduce its reliability, impacting the stretch reflex [6]. Our exploratory analyses revealed lower CP values in participants with absent ankle reflex, being less disturbed by vibration than other participants, but the differences were not significant (*p* ≥ 0.56). Vibration may not be an appropriate method to highlight post-chemotherapy sensory coping strategies for reduced postural control.

Chemotherapy-induced postural instability may affect activities of daily living, particularly those involving dual tasks. This study showed an increase in postural instability under DT conditions, probably because of an increase in cognitive interference [18] and inability to use vision for stabilization, busy writing the message.

In contrast, dynamic stability, which is also relevant to daily life, was not changed post chemotherapy, a finding that differs from the results of a previous study [48]. Methodological difficulties related to the understanding and execution of the tests, fatigue, and the high inter-participant variability, as reported previously [12, 49], may have affected the findings [49] and further studies are needed to provide more information on the effect of chemotherapy on limits of stability.

Given the decrease in postural control in conditions that primarily rely on somatosensory and vestibular inputs [8], the sensory impairments, and the poorer neurotoxicity sub-scale scores on the quality of life questionnaire beyond the minimal clinically significant difference [50], we assumed that somatosensory deficits might be associated with postural instability. However, only few CP and GRF parameters were moderately correlated with the mTNS score in exploratory analysis (*supplementary material 1*), corroborating a previous study [14], and not with the NTx score. Furthermore, contrary to our hypothesis, there were no differences in postural control disorders between the CIPN severity subgroups. Thus, greater severity of CIPN does not appear to lead to greater deterioration in postural control.

An explanation, supported by several studies [1, 4, 51, 52] and the elevated baseline CP values in this study, is that other factors were also involved in the alteration of postural control. Physical inactivity [14, 51], vestibular deficits [6], or previous treatments, such as mastectomy [53], could play an important role in the development of postural disorders. Moreover, cognitive impairments [14], such as attentional deficits linked to anxiety and fatigue [1], or proximal proprioceptive deficits preventing accurate force generation [14, 51, 52] may have contributed to the postural impairment.

### Limitations

Selection bias favored inclusion of people with breast cancer, reducing the external validity of the study. Only one assessment was performed pre and post chemotherapy. Repeated measurements would have been preferable because of the high variability of test results identified in a previous study [54]. Despite the random order of test conditions and the breaks between trials, the 75-minute duration of the evaluations may have caused fatigue that could have affected the results [55]. Due to clinical constraints, some tests (five pre, four post) exceeded the one-month pre- and post-treatment interval, which could have influenced the results (the longer the period, the more divergent the results) [51]. According to exploratory analyses, the effects of fatigue and time interval appeared to be minimal.

### Perspectives

The high variability of the results highlights the heterogeneity of postural control impairment after chemotherapy. Many factors in addition to somatosensory deficits may impact postural control. According to our exploratory analyses (*supplementary material 1*), physical inactivity seemed to influence postural control disorders and should be evaluated with more objective validated measures in future studies. Individual assessment of previous treatments, fatigue, cognitive impairments, and comorbidities will also help to clarify the factors involved in impaired postural control.

### Practical implications and conclusions

In conclusion, given the severity of impairment of postural control found, we suggest that postural control should be systematically evaluated with eyes closed on hard surface in women with breast cancer to identify chemotherapy-induced postural impairment. With a force platform, CP total and AP length and RMS with ML max GRF could be used to observe postural control changes. In a clinical setting without the use of force or stabilometric platforms, the literature recommends functional balance tests such as the Fullerton Advanced Balance Scale to assess postural control [10, 56]. Based on what has been observed, supportive physical activity care could include sensorimotor exercises with eyes closed and dual task exercises to limit postural control disorders after chemotherapy. Sensorimotor exercises programs revealed to be efficient in preventing postural control impairment in patients treated with taxanes for breast cancer [57], and with vinca-alkaloids or oxaliplatin for lymphoma and colorectal cancers [58]. They also may have a positive impact on preventing and treating CIPN sign and symptoms which encourage their use in practice [59, 60]. Further high-quality studies are required to better define their effects and formulate recommendations [61].

## Electronic supplementary material

Below is the link to the electronic supplementary material.

Supplementary Material 1

## Data Availability

Data will be made available in the Yareta repository.
