# Supplementary material for: Postural control impairments following neurotoxic chemotherapy in women with cancer: a prospective observational study
Source: BMC Womens Health. 2025 May 30;25:269. doi: 10.1186/s12905-025-03790-4 (PMC12124009; doi:10.1186/s12905-025-03790-4)
Supplement: Supplementary file 1 — Supplementary Material 1 [file 12905_2025_3790_MOESM1_ESM.docx]

*Supplementary material*

| Modified Total Neuropathy Score | | | | |
| --- | --- | --- | --- | --- |
|  | **Pre** | **Post** | **Difference** | **Corrected *p*-value** |
| Sensory symptoms (/4) | 0.24 ± 0.44 (0 – 1) | 0.97 ± 0.85 (0 – 3) | 0.73 ± 0.88 (0 – 3) | **≤0.001** |
| Motor symptoms (/4) | 0.55 ± 0.56 (0 – 2) | 1.49 ± 0.71 (0 – 3) | 0.94 ± 0.70 (0 – 3) | **≤0.001** |
| Superficial sensitivity (/4) | 0.03 ± 0.17 (0 – 1) | 0.55 ± 0.75 (0 – 2) | 0.52 ± 0.76 (0 – 2) | **≤0.001** |
| Vibratory sensitivity (/4) | 0.24 ± 0.87 (0 – 4) | 1.33 ± 1.47 (0 – 4) | 1.09 ± 1.40 (-1 – 4) | **≤0.001** |
| Reflex sensitivity (/4) | 0.21 ± 0.42 (0 – 1) | 2.12 ± 1.27 (0 – 4) | 1.91 ± 1.23 (0 – 4) | **≤0.001** |
| Strength (/4) | 0.24 ± 0.50 (0 – 2) | 0.76 ± 0.66 (0 – 3) | 0.52 ± 0.57 (0 – 2) | **≤0.001** |
| Total score (/24) | 1.52 ± 1.54 (0 – 8) | 7.21 ± 3.60 (1 – 16) | 5.70 ± 3.17 (1 – 14) | **≤0.001** |
| Grade severity  1  2  3  4 | 33 (100)  0  0  0 | 20 (61)  13 (39)  0  0 | -13  +13  0  0 | - |

***Table 1.*** *mTNS results. Data presented as mean ± SD (Min-Max) or n (%). mTNS = modified Total Neuropathy Score. N = 33*

| Eyes open condition on foam (EOF) | | | | | Eyes closed with vibration condition (ECV) | | | | Dual task condition (DT) | | | |
| --- | --- | --- | --- | --- | --- | --- | --- | --- | --- | --- | --- | --- |
|  | **Pre** | **Post** | **Difference** | ***p*-value** | **Pre** | **Post** | **Difference** | ***p*-value** | **Pre** | **Post** | **Difference** | ***p*-value** |
| CP | | | |  |  |  |  |  |  |  |  |  |
| Planar | | | |  |  |  |  |  |  |  |  |  |
| Length (mm) | 594.60 ± 185.55 (376.57 – 1156.90) | 668.00 ± 214.91 (362.31 – 1288.95) | 73.40 ± 144.52 (-453.27 – 344.98) | **0.020** | 616.81 ± 248.59 (331.79 – 1348.31) | 682.05 ± 266.39 (340.84 – 1123.87) | 65.24 ± 193.84 (-372.35 – 388.92) | 0.211 | 384.69 ± 93.94 (222.29 – 590.63) | 447.26 ± 111.67 (283.10 – 711.43) | 62.57 ± 59.69 (-37.61 – 226.26) | **≤0.001** |
| Area (mm^2^) | 578.79 ± 479.95 (171.95 – 2324.93) | 523.41 ± 374.54 (205.03 – 2359.47) | -55.38 ± 357.20 (-1673.1 – 290.29) | 0.568 | 522.70 ± 580.04 (89.66 – 2979.88) | 560.27 ± 522.42 (114.26 – 2303.72) | 37.56 ± 372.53 (-1075.76 – 1021.63) | 0.709 | 185.80 ± 138.37 (62.98 – 692.22) | 260.97 ± 161.44 (71.22 – 768.84) | 75.17 ± 134.56 (-195.27 – 479.45) | **0.005** |
| Maximal speed (mm/s) | 103.35 ± 30.97 (62.43 – 191.21) | 118.98 ± 44.60 (65.65 – 242.81) | 15.63 ± 31.52 (-83.14 – 75.23) | **0.020** | 107.12 ± 47.68 (47.02 – 256.70) | 123.38 ± 60.04 (53.86 – 252.75) | 16.26 ± 44.63 (-54.86 – 149.49) | 0.291 | 67.46 ± 22.42 (37.01 – 143.57) | 73.90 ± 21.34 (46.10 – 125.31) | 6.43 ± 24.94 (-78.37 – 53.65) | 0.148 |
| RMS (mm) | 1.33 ± 0.45 (0.80 – 2.66) | 1.50 ± 0.52 (0.79 – 3.07) | 0.17 ± 0.34 (-1.02 – 0.72) | **0.013** | 1.36 ± 0.62 (0.67 – 3.24) | 1.54 ± 0.71 (0.66 – 3.01) | 0.19 ± 0.49 (-0.67 – 1.09) | 0.211 | 0.80 ± 0.24 (0.48 – 1.32) | 0.93 ± 0.26 (0.53 – 1.50) | 0.14 ± 0.19 (-0.43 – 0.49) | **≤0.001** |
| ML | | | |  |  |  |  |  |  |  |  |  |
| Length (mm) | 349.19 ± 109.49 (222.20 – 692.60) | 376.59 ± 110.75 (210.46 – 724.11) | 27.40 ± 69.96 (-239.56 – 153) | 0.072 | 303.44 ± 111. 91 (153.78 – 559.13) | 331.75 ± 133.76 (172.43 – 605.06) | 28.31 ± 96.68 (-163.36 – 220.54) | 0.211 | 200.06 ± 57.82 (101.23 – 369.14) | 226.45 ± 68.97 (137.79 – 421.96) | 26.39 ± 34.47 (-19.56 – 113.56) | **0.001** |
| RMS (mm) | 1.02 ± 0.34 (0.63 – 2.06) | 1.08 ± 0.33 (0.63 – 2.19) | 0.07 ± 0.21 (-0.72 – 0.45) | 0.160 | 0.86 ± 0.35 (0.40 – 1.68) | 0.96 ± 0.44 (0.46 – 2.01) | 0.10 ± 0.33 (-0.50 – 0.83) | 0.211 | 0.55 ± 0.18 (0.29 – 1.06) | 0.62 ± 0.21 (0.37 – 1.21) | 0.07 ± 0.14 (-0.45 – 0.37) | **0.010** |
| AP | | | |  |  |  |  |  |  |  |  |  |
| Length (mm) | 408.12 ± 129.98 (245.68 – 795.44) | 471.80 ± 164.48 (249.05 – 924.70) | 63.68 ± 117.22 (-334.54 – 288.67) | **0.006** | 468.63 ± 206.06 (261.29 – 1116.93) | 522.77 ± 213.27 (251.31 – 934.61) | 54.14 ± 160.82 (-300.60 – 353.16) | 0.211 | 285.17 ± 68.28 (183.32 – 432.06) | 337.27 ± 82.54 (210.13 – 513.72) | 52.10 ± 47.86 (-31.93 – 169.77) | **≤0.001** |
| RMS (mm) | 1.19 ± 0.40 (0.68 – 2.34) | 1.39 ± 0.50 (0.69 – 2.78) | 0.20 ± 0.35 (-0.98 – 0.9) | **0.013** | 1.34 ± 0.62 (0.69 – 3.31) | 1.51 ± 0.68 (0.66 – 3.09) | 0.17 ± 0.50 (-0.84 – 1.18) | 0.211 | 0.78 ± 0.22 (0.50 – 1.35) | 0.92 ± 0.24 (0.54 – 1.40) | 0.14 ± 0.17 (-0.23 – 0.49) | **≤0.001** |
| GRF | | | |  |  |  |  |  |  |  |  |  |
| V | | | |  |  |  |  |  |  |  |  |  |
| Max values (Kg) | 10.01 ± 0.27 (9.38 – 10.59) | 10.39 ± 0.58 (9.18 – 11.52) | 0.37 ± 0.56 (-0.79 – 1.64) | **0.006** | 9.80 ± 0.27 (9.28 – 10.41) | 10.13 ± 0.57 (8.94 – 11.35) | 0.34 ± 0.51 (-0.69 – 1.59) | **0.041** | 9.78 ± 0.28 (9.23 – 10.37) | 10.13 ± 0.64 (8.71 – 11.39) | 0.36 ± 0.55 (-0.85 – 1.45) | **0.003** |
| Variability (Kg) | 0.04 ± 0.02 (0.01 – 0.09) | 0.05 ± 0.03 (0.02 – 0.13) | 0.01 ± 0.01 (-0.01 – 0.05) | **0.003** | 0.01 ± 0.01 (0.01 – 0.03) | 0.01 ± 0.01 (0.01 – 0.03) | 0.00 ± 0.01 (-0.02 – 0.01) | 0.562 | 0.01 ± 0.01 (0.01 – 0.04) | 0.02 ± 0.01 (0.01 – 0.04) | 0.00 ± 0.01 (-0.02 – 0.01) | 0.118 |
| ML | | | |  |  |  |  |  |  |  |  |  |
| Max values (Kg) | 2.20 ± 1.50 (0.35 – 7.05) | 2.73 ± 1.73 (0.61 – 8.01) | 0.53 ± 1.06 (-1.67 – 3.33) | **0.042** | 1.77 ± 1.74 (-0.34 – 7.46) | 2.45 ± 2.13 (-0.27 – 7.79) | 0.69 ± 1.84 (-2.44 – 5.47) | 0.248 | 0.19 ± 0.71 (-0.72 – 1.74) | 0.67 ± 0.95 (-1.16 – 2.49) | 0.48 ± 0.67 (-0.93 – 1.76) | **0.006** |
| Variability (Kg) | 1.08 ± 0.59 (0.52 – 3.37) | 1.24 ± 0.62 (0.56 – 3.51) | 0.16 ± 0.20 (-0.23 – 0.53) | **0.006** | 0.98 ± 0.48 (0.38 – 2.28) | 1.19 ± 0.63 (0.39 – 2.53) | 0.21 ± 0.41 (-0.31 – 1.34) | 0.106 | 0.55 ± 0.27 (0.19 – 1.29) | 0.69 ± 0.33 (0.23 – 1.35) | 0.15 ± 0.15 (-0.04 – 0.48) | **≤0.001** |
| AP | | | |  |  |  |  |  |  |  |  |  |
| Max values (Kg) | 7.24 ± 3.42 (3.72 – 18.83) | 8.28 ± 4.01 (3.24 – 19.43) | 1.04 ± 4.39 (-11.30 – 13.14) | 0.201 | 8.58 ± 6.97 (3.02 – 38.31) | 9.17 ± 6.33 (2.58 – 30.61) | 0.59 ± 9.27 (-30.98 – 22.96) | 0.513 | 8.36 ± 10.56 (2.37 – 40.72) | 6.22 ± 3.94 (2.52 – 21.98) | -2.14 ± 11.64 (-36.27 – 18.96) | 0.391 |
| Variability (Kg) | 1.30 ± 0.68 (0.68 – 3.94) | 1.63 ± 0.86 (0.67 – 4.70) | 0.33 ± 0.35 (-0.13 – 1.18) | **0.003** | 1.43 ± 0.76 (0.70 – 3.16) | 1.88 ± 1.20 (0.53 – 5.12) | 0.46 ± 0.76 (-0.62 – 2.45) | 0.066 | 0.80 ± 0.40 (0.40 – 1.60) | 1.07 ± 0.46 (0.46 – 2.12) | 0.28 ± 0.18 (-0.04 – 0.60) | **≤0.001** |

***Table 2.*** *CP and GRF parameters in EOF, CRV, and DT conditions. Data presented as mean ± SD (Min-Max). AP = anteroposterior, CP = center of pressure, GRF = Ground Reaction Force, ML = medio-lateral, V = vertical. Corrected p-values*

| Eyes closed condition (EC) n = 33 for CP parameters. n = 24 for ML and AP GRF. n = 31 for V GRF parameters | | | | | | | | | |
| --- | --- | --- | --- | --- | --- | --- | --- | --- | --- |
|  | **Pre** | | **Post** | | **Difference** | | ***p-*value T1 vs. T2** | | ***p*-value difference**  **mTNS 1 vs. mTNS 2** |
| Severity | **mTNS 1 (N = 20)** | **mTNS 2 (N = 13)** | **mTNS 1** | **mTNS 2** | **mTNS 1** | **mTNS 2** | **mTNS 1** | **mTNS 2** |  |
| CP | | | | | | | | | |
| Planar | | | | | | | | | |
| Total length (mm) | 433.74 ± 121.95 (303.78 – 786.13) | 569.66 ± 312.19 (298.14 – 1340.21) | 543.64 ± 199.88 (308.80 – 1012.15) | 709.42 ± 312.69 (376.25 – 1548.88) | 101.54 ± 111.88 (-18.92 – 407.19) | 139.77 ± 141.57 (-246.21 – 358.64) | **0.001** | **0.036** | 0.176 |
| Maximal speed (mm/s) | 84.81 ± 31.68 (42.99 – 169.77) | 92.65 ± 49.45 (39.50 – 228.24) | 96.91 ± 42.66 (42.99 – 234.07) | 125.80 ± 57.42 (57.91 – 260.66) | 11.51 ± 27.98 (-25.70 – 89.09) | 33.14 ± 23.12 (-8.54 – 68.16) | 0.140 | **0.012** | 0.089 |
| RMS (mm) | 0.97 ± 0.34 (0.56 – 1.88) | 1.22 ± 0.72 (0.58 – 2.95) | 1.23 ± 0.61 (0.58 – 3.15) | 1.67 ± 0.85 (0.75 – 3.80) | 0.25 ± 0.38 (-0.12 – 1.63) | 0.45 ± 0.27 (0.04 – 0.85) | **0.002** | **0.012** | 0.089 |
| ML | | | | | | | | | |
| Length (mm) | 224.65 ± 76.25 (133.51 – 442.60) | 320.66 ± 197.60 (147.24 – 839.95) | 284.72 ± 147.28 (133.51 – 637.98) | 356.79 ± 229.20 (191.79 – 1046.55) | 55.00 ± 92.36 (-21.72 – 386.10) | 36.12 ± 106.37 (-219.37 – 206.60) | **0.004** | 0.124 | 0.971 |
| RMS (mm) | 0.63 ± 0.24 (0.35 – 1.33) | 0.95 ± 0.65 (0.40 – 2.58) | 0.84 ± 0.58 (0.35 – 2.95) | 1.08 ± 0.76 (0.50 – 3.39) | 0.19 ± 0.42 (-0.14 – 1.83) | 0.13 ± 0.35 (-0.63 – 0.81) | **0.014** | 0.124 | 0.971 |
| AP | | | | | | | | | |
| Length (mm) | 323.18 ± 85.79 (222.63 – 563.79) | 399.26 ± 201.11 (222.08 – 867.33) | 398.91 ± 123.39 (235.68 – 637.98) | 534.83 ± 189.93 (282.23 – 926.06) | 70.10 ± 64.57 (-8.27 – 221.54) | 135.58 ± 96.96 (-73.99 – 261.58) | **0.001** | **0.012** | 0.098 |
| RMS (mm) | 0.94 ± 0.31 (0.58 – 1.71) | 1.11 ± 0.57 (0.57 – 2.42) | 1.15 ± 0.41 (0.60 – 1.91) | 1.56 ± 0.63 (0.77 – 2.77) | 0.20 ± 0.20 (-0.04 – 0.60) | 0.45 ± 0.28 (0.16 – 1.14) | **0.002** | **0.012** | 0.064 |
| GRF | | | | | | | | | |
| V | | | | | | | | | |
| Max values (Kg) | 9.74 ± 0.27 (9.24 – 10.36) | 9.80 ± 0.24 (9.16 – 10.06) | 10.05 ± 0.66 (8.77 – 11.27) | 10.12 ± 0.47 (9.25 – 11.09) | 0.31 ± 0.54 (-0.96 – 1.25) | 0.32 ± 0.50 (-0.48 – 1.18) | 0.054 | 0.102 | 0.780 |
| Variability (Kg) | 0.01 ± 0.00 (0.01 – 0.02) | 0.02 ± 0.02 (0.01 – 0.08) | 0.01 ± 0.00 (0.01 – 0.02) | 0.01 ± 0.01 (0.01 – 0.03) | 0.00 ± 0.00 (-0.00 – 0.01) | -0.00 ± 0.02 (-0.05 – 0.01) | **0.033** | 0.724 | 0.684 |
| ML |  |  |  |  |  |  |  |  |  |
| Max values (Kg) | 0.99 ± 1.77 (-1.02 – 6.25) | 1.50 ± 2.24 (-0.51 – 5.60) | 2.05 ± 3.26 (-0.49 – 12.08) | 2.78 ± 3.26 (-0.22 – 9.41) | 1.06 ± 2.45 (-1.24 – 9.13) | 1.28 ± 1.42 (-0.18 – 4.39) | 0.098 | **0.026** | 0.558 |
| Variability (Kg) | 0.72 ± 0.51 (0.27 – 2.21) | 0.86 ± 0.54 (0.32 – 1.83) | 0.99 ± 0.81 (0.24 – 3.16) | 1.14 ± 0.73 (0.42 – 2.78) | 0.28 ± 0.50 (-0.09 – 1.91) | 0.28 ± 0.33 (0 – 1.05) | **0.033** | **0.026** | 0.682 |
| AP |  |  |  |  |  |  |  |  |  |
| Max values (Kg) | 5.94 ± 3.66 (2.63 – 14.93) | 5.93 ± 2.41 (3.63 – 10.87) | 6.49 ± 2.85 (2.79 – 11.44) | 7.53 ± 4.28 (3.58 – 18.32) | 0.56 ± 1.63 (-4.14 – 2.96) | 1.60 ± 4.08 (-5.06 – 10.20) | 0.054 | 0.313 | 0.558 |
| Variability (Kg) | 1.11 ± 0.65 (0.42 – 2.79) | 0.98 ± 0.49 (0.38 – 1.88) | 1.40 ± 0.79 (0.55 – 3.15) | 1.66 ± 0.89 (0.66 – 3.68) | 0.30 ± 0.29 (-0.03 – 1.06) | 0.68 ± 0.45 (0.28 – 1.80) | **0.012** | **0.026** | **0.040** |

***Table 3****. CP and GRF parameters in EC condition in subgroups of severity of TNSm. Data are mean ± SD (Min-Max). AP = anteroposterior, CP = center of pressure, GRF = Ground Reaction Force, ML = medio-lateral, mTNS = modified Total Neuropathy Score, V = vertical. Corrected p-values*

| **Correlation heatmap** | | | | | | | | |
| --- | --- | --- | --- | --- | --- | --- | --- | --- |
|  | **EC** | | | | **ECF** | | | |
| **Parameter** |  | FACT-NTx (/44) | mTNS score (/24) | Physical activity from 3 to 6 MET (hour/week) |  | FACT-NTx (/44) | mTNS score (/24) | Physical activity from 3 to 6 MET (hour/week) |
| **Total CP length (mm)** | Correlations coefficient | -0.08 | 0.27 | -0.07 | Correlations coefficient | -0.08 | 0.27 | **-0.50** |
|  | *p*-value | 0.918 | 0.206 | 0.804 | *p*-value | 0.884 | 0.189 | **0.010** |
| **Max speed (mm/s)** | Correlations coefficient | -0.32 | **0.46** | -0.01 | Correlations coefficient | -0.37 | 0.35 | **-0.41** |
|  | *p*-value | 0.233 | **0.035** | 0.946 | *p*-value | 0.350 | 0.087 | **0.023** |
| **RMS (mm)** | Correlations coefficient | -0.17 | 0.35 | -0.12 | Correlations coefficient | -0.22 | 0.35 | **-0.47** |
|  | *p*-value | 0.710 | 0.125 | 0.804 | *p*-value | 0.444 | 0.087 | **0.010** |
| **AP CP length (mm)** | Correlations coefficient | -0.06 | 0.26 | -0.11 | Correlations coefficient | -0.16 | 0.34 | **-0.51** |
|  | *p*-value | 0.734 | 0.206 | 0.804 | *p*-value | 0.640 | 0.087 | **0.010** |
| **AP RMS (mm)** | Correlations coefficient | -0.22 | 0.39 | -0.16 | Correlations coefficient | -0.22 | 0.37 | **-0.49** |
|  | *p*-value | 0.550 | 0.08 | 0.804 | *p*-value | 0.444 | 0.087 | **0.010** |
| **ML CP length (mm)** | Correlations coefficient | 0.04 | 0.05 | 0.11 | Correlations coefficient | -0.01 | 0.20 | **-0.47** |
|  | *p*-value | 0.922 | 0.793 | 0.804 | *p*-value | 0.963 | 0.308 | **0.010** |
| **ML RMS (mm)** | Correlations coefficient | 0.00 | 0.05 | 0.06 | Correlations coefficient | -0.05 | 0.20 | **-0.45** |
|  | *p*-value | 0.992 | 0.793 | 0.804 | *p*-value | 0.893 | 0.308 | **0.014** |
| **ML max GRF (Kg)** | Correlations coefficient | -0.52 | 0.31 | 0.15 | Correlations coefficient | -0.08 | 0.17 | -0.19 |
|  | *p*-value | 0.100 | 0.206 | 0.804 | *p*-value | 0.884 | 0.416 | 0.372 |
| **V max GRF (Kg)** | Correlations coefficient | -0.09 | 0.17 | 0.09 | Correlations coefficient | -0.23 | 0.35 | -0.18 |
|  | *p*-value | 0.918 | 0.458 | 0.804 | *p*-value | 0.444 | 0.087 | 0.372 |
| **AP variability GRF (Kg)** | Correlations coefficient | -0.45 | **0.59** | -0.18 | Correlations coefficient | -0.35 | 0.49 | **-0.55** |
|  | *p*-value | 0.135 | **0.020** | 0.804 | *p*-value | 0.444 | 0.087 | **0.010** |

|  |  |  |  |  |  |  |  |  |  |  |
| --- | --- | --- | --- | --- | --- | --- | --- | --- | --- | --- |
| **1.00** | **0.80** | **0.60** | **0.40** | **0.20** | **0.00** | **-0.20** | **-0.40** | **-0.60** | **-0.80** | **1.00** |
| Spearman correlation coefficient (*r*) | | | | | | | | | | |

***Table 4.*** *Correlation heatmap between CP and GRF parameters differences (T2-T1) and FACT-NTx, mTNS and physical activity post chemotherapy (T2) in EC and ECF conditions assessed by Spearman correlations. AP = anteroposterior, FACT-NTx = Functional Assessment of Cancer Therapy-Neurotoxicity, GRF = ground reaction force, ML = mediolateral, mTNS = modified Total Neuropathy Score, RMS = root mean square, V = vertical. In bold, significant correlations (p < 0.05). Parameters and conditions were those most affected after chemotherapy. Corrected p-values*

| **Subgroups comparisons** | | | | | | | | | |
| --- | --- | --- | --- | --- | --- | --- | --- | --- | --- |
|  |  | **EC** | | | | **ECF** | | | |
|  |  | **Treatment change** | | **Fatigue** | | **Treatment change** | | **Fatigue** | |
|  | **Parameter** | **No** | **Yes** | **< 70 / 100** | ≥ **70 / 100** | **No** | **Yes** | **< 70 / 100** | ≥ **70 / 100** |
| **Total CP length (mm)** | **Subgroups values** | 101.73 ± 127.96 | 150.81 ± 111.98 | 130.72 ± 101.36 | 84.13 ± 166.24 | 295.85 ± 366.20 | 656.30 ± 447.19 | 455.30 ± 417.42 | 289.56 ± 425.69 |
|  | **Between groups *p*-value** | 0.511 | | 0.814 | | 0.076 | | 0.491 | |
| **Max speed (mm/s)** | **Subgroups values** | 16.17 ± 24.79 | 28.92 ± 33.87 | 13.89 ± 23.79 | 34.16 ± 32.76 | 36.54 ± 68.12 | 145.92 ± 108.26 | 64.95 ± 94.79 | 80.55 ± 100.87 |
|  | **Between groups *p*-value** | 0.652 | | 0.424 | | 0.076 | | 0.845 | |
| **RMS (mm)** | **Subgroups values** | 0.26 ± 0.27 | 0.46 ± 0.48 | 0.31 ± 0.28 | 0.36 ± 0.49 | 0.63 ± 0.97 | 1.71 ± 1.22 | 1.07 ± 1.18 | 0.68 ± 1.07 |
|  | **Between groups *p*-value** | 0.511 | | 0.814 | | 0.076 | | 0.571 | |
| **AP CP length (mm)** | **Subgroups values** | 84.99 ± 86.10 | 120.96 ± 77.25 | 107.98 ± 85.98 | 68.11 ± 76.09 | 235.21 ± 274.39 | 540.83 ± 383.51 | 364.67 ± 329.65 | 243.06 ± 355.49 |
|  | **Between groups *p*-value** | 0.511 | | 0.814 | | 0.076 | | 0.571 | |
| **AP RMS (mm)** | **Subgroups values** | 0.26 ± 0.22 | 0.38 ± 0.32 | 0.32 ± 0.29 | 0.25 ± 0.19 | 0.64 ± 0.87 | 1.71 ± 1.23 | 1.06 ± 1.08 | 0.74 ± 1.15 |
|  | **Between groups *p*-value** | 0.511 | | 0.814 | | 0.076 | | 0.590 | |
| **ML CP length (mm)** | **Subgroups values** | 40.09 ± 87.77 | 64.75 ± 118.77 | 51.82 ± 67.34 | 37.78 ± 148.90 | 146.39 ± 197.63 | 283.56 ± 188.33 | 213.52 ± 207.40 | 129.16 ± 186.71 |
|  | **Between groups *p*-value** | 0.969 | | 0.814 | | 0.076 | | 0.491 | |
| **ML RMS (mm)** | **Subgroups values** | 0.13 ± 0.28 | 0.26 ± 0.57 | 0.15 ± 0.24 | 0.20 ± 0.63 | 0.37 ± 0.74 | 0.91 ± 0.66 | 0.62 ± 0.82 | 0.33 ± 0.54 |
|  | **Between groups *p*-value** | 0.969 | | 0.814 | | 0.076 | | 0.491 | |
| **ML max GRF** | **Subgroups values** | 0.74 ± 1.38 | 2.11 ± 3.18 | 0.92 ± 1.36 | 1.68 ± 3.37 | 2.61 ± 3.82 | 4.02 ± 2.97 | 3.89 ± 3.81 | 0.91 ± 1.83 |
|  | **Between groups *p*-value** | 0.511 | | 0.814 | | 0.191 | | 0.491 | |
| **V max GRF (Kg)** | **Subgroups values** | 0.36 ± 0.56 | 0.22 ± 0.42 | 0.21 ± 0.54 | 0.57 ± 0.35 | 0.51 ± 0.69 | 0.67 ± 0.56 | 0.45 ± 0.68 | 0.84 ± 0.49 |
|  | **Between groups *p*-value** | 0.729 | | 0.424 | | 0.642 | | 0.491 | |
| **AP variability GRF (Kg)** | **Subgroups values** | 0.37 ± 0.29 | 0.61 ± 0.57 | 0.47 ± 0.44 | 0.37 ± 0.25 | 0.78 ± 1.10 | 2.04 ± 1.46 | 1.33 ± 1.21 | 0.70 ± 1.56 |
|  | **Between groups *p*-value** | 0.541 | | 0.814 | | 0.076 | | 0.491 | |

***Table 5.*** *Subgroups comparisons of CP and GRF parameters differences (T2-T1) in EC and ECF conditions assessed by Mann Whitney tests. Cut-off of fatigue based on 75^th^ distribution percentile. Non-corrected p-values*
